# Computational Exploration of Licorice for Lead Compounds against *Plasmodium vivax* Duffy Binding Protein Utilizing Molecular Docking and Molecular Dynamic Simulation

**DOI:** 10.3390/molecules28083358

**Published:** 2023-04-11

**Authors:** Muhammad Yasir, Jinyoung Park, Eun-Taek Han, Won Sun Park, Jin-Hee Han, Yong-Soo Kwon, Hee-Jae Lee, Wanjoo Chun

**Affiliations:** 1 Department of Pharmacology, Kangwon National University School of Medicine, Chuncheon 24341, Republic of Korea; 2 Department of Medical Environmental Biology and Tropical Medicine, Kangwon National University School of Medicine, Chuncheon 24341, Republic of Korea; 3 Department of Physiology, Kangwon National University School of Medicine, Chuncheon 24341, Republic of Korea; 4 College of Pharmacy, Kangwon National University School of Medicine, Chuncheon 24341, Republic of Korea

**Keywords:** *Plasmodium vivax*, molecular docking, molecular dynamic simulation, DBP inhibition

## Abstract

*Plasmodium vivax* (*P. vivax*) is one of the human’s most common malaria parasites. *P. vivax* is exceedingly difficult to control and eliminate due to the existence of extravascular reservoirs and recurring infections from latent liver stages. Traditionally, licorice compounds have been widely investigated against viral and infectious diseases and exhibit some promising results to combat these diseases. In the present study, computational approaches are utilized to study the effect of licorice compounds against *P. vivax* Duffy binding protein (DBP) to inhibit the malarial invasion to human red blood cells (RBCs). The main focus is to block the DBP binding site to Duffy antigen receptor chemokines (DARC) of RBC to restrict the formation of the DBP–DARC complex. A molecular docking study was performed to analyze the interaction of licorice compounds with the DARC binding site of DBP. Furthermore, the triplicates of molecular dynamic simulation studies for 100 ns were carried out to study the stability of representative docked complexes. The leading compounds such as licochalcone A, echinatin, and licochalcone B manifest competitive results against DBP. The blockage of the active region of DBP resulting from these compounds was maintained throughout the triplicates of 100 ns molecular dynamic (MD) simulation, maintaining stable hydrogen bond formation with the active site residues of DBP. Therefore, the present study suggests that licorice compounds might be good candidates for novel agents against DBP-mediated RBC invasion of *P. vivax*.

## 1. Introduction

Malaria, an infectious disease, has posed a significant threat to human health. [1]. There are nine unique species of the unicellular eukaryotic parasite *Plasmodium* that infect humans, which comprise *P. falciparum*, *P. vivax*, *P. malariae*, *P. ovale curtisi*, *P. ovale wallikeri* [2], *P. knowlesi*, *P. cynomolgi* [3], *P. simium* [4,5], and *P. brasilianum* [6]. Only two of the nine *Plasmodium* species, *P. falciparum* and *P. vivax*, appear to constitute a significant threat to the global malaria burden. Around 80% of the global *P. vivax* burden is carried by Asia and the Asia-Pacific area. In these areas, *P. vivax* is by far the most prevalent cause of human malaria [7]. The proportion of malaria caused by *P. vivax* has increased in regions where the *P. falciparum* load has been lowered by vigorous malaria control strategies [8]. To treat the disease, quinine-like drugs (such as the cinchona alkaloid quinine, 4-aminoquinoline chloroquine (CQ), hydroxychloroquine, primaquine, and pyronaridine) and artemisinin-based combination therapy are employed. However, drug resistance has evolved, presumably as a result of mutations in the active regions of drug targets or biochemical alterations in drug receptors, necessitating the development of innovative techniques [9].

*Plasmodium* sporozoite remains in the liver and matures into merozoite to invade human RBCs in the blood stage. *P. vivax* infiltrates human RBCs by engaging a protein known as Duffy binding protein (DBP). *P. vivax* DBP interacts with the Duffy antigen receptor for chemokines on the surface of red blood cells (DARC). DARC is a transmembrane, glycosylated protein of approximately 35–40 kDa that is found on chromosome 1 (1.q22-1.q23). It has an intracellular C-terminal domain and an extracellular N-terminal region that includes chemokine binding receptors [10]. *Plasmodium* releases micronemes and rhoptries, two specialized apical organelles, to enter an RBC. DBP binds DARC with great affinity via Duffy binding-like domains and localizes to micronemes. DBP is a promising vaccine and therapeutic option for *P. vivax* malaria [11].

Herbs have long been utilized in traditional medicine and include a wide range of phytochemical components including terpenoids, phenols, lignins, stilbenes, tannins, flavonoids, quinones, coumarins, alkaloids, amines, betalains, and other metabolites [12]. The success of two plant-based antimalarial medications, quinine and artemisinin, prompted researchers to look for antimalarial compounds from a variety of botanic sources. Without a doubt, licorice is one of the most popular medicinal herbs [13]. Glycyrrhiza glabra is 1 of roughly 30 species in the genus *Glycyrrhiza* L. *Glycyrrhiza glabra* L. is a traditional medicinal herb that grows all over the world. [14]. Ancient historical literature from China, India, and Greece references its application to treat hepatic, viral, and respiratory tract diseases [15]. *Glycyrrhiza glabra* has been discovered to have a potential therapeutic effect on *P. falciparum* [16]. Given the rising resistance of *Plasmodium* strains to well-known anti-malarial treatments [17], the development of novel anti-malarial therapies is in high demand.

Computer-aided drug design (CADD) has become an increasingly important tool in the field of drug discovery and development. The use of computational methods and software allows for the rapid and efficient screening of large numbers of compounds, reducing the time and cost associated with traditional experimental methods [18]. CADD allows for the creation of detailed models of proteins, enzymes, and other biological molecules, providing a deeper understanding of the underlying biology [19]. This can be used to identify new drug targets and optimize the properties of existing drugs. In this work, computational approaches are used to investigate the therapeutic potential of licorice compounds against the DARC binding domain of *P. vivax* DBP. Therefore, the literature survey was performed to screen the best and most recently reported biologically active licorice compounds. Molecular docking studies predict five compounds to be potent against DBP. Furthermore, molecular dynamic modeling studies were carried out to predict the best compounds and examine the sustainability of the docking complexes based on the efficacy of DBP binding site blockage.

## 2. Results

**Figure 1:**
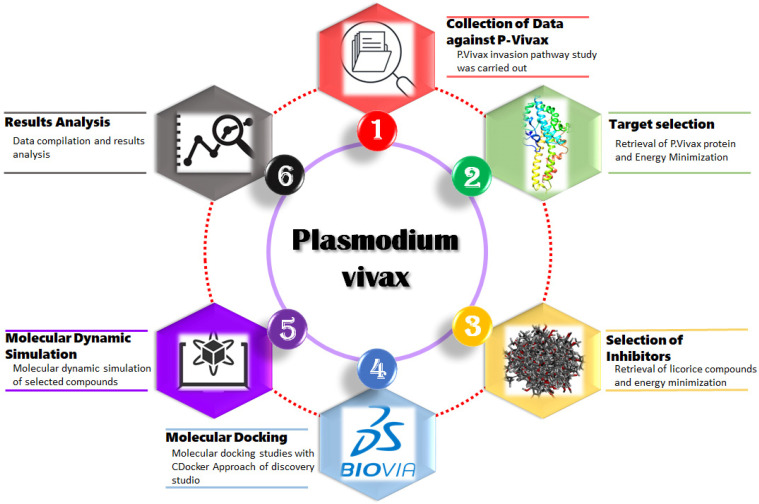
Workflow diagram for the screening of licorice inhibitors against DBP.

Figure 1 depicts the suggested research workflow. To anticipate and observe the interactions of licorice compounds with DBP protein, six stages were taken, including protein retrieval, retrieval of best licorice compounds, molecular docking, MD simulation experiments, and data analysis.

### 2.1. Structural Assessment of P. vivax DBP

**Figure 2:**
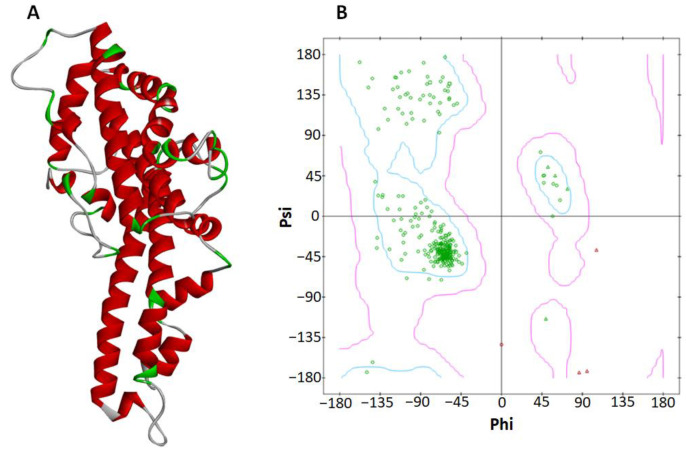
The 3D structure of *P. vivax* DBP after energy minimization is shown on the right (**A**). The (**B**) Ramachandran graph exhibiting that all of the amino acid residues are in the permitted zone and there are no outliers.

*P. vivax* DBP has 317 amino acids, 62% of which are helices, 1% are sheets, 36% are coils, and 22% are turns. *P. vivax* DBP Ramachandran plots show that 95.8% of all residues were in favorable areas. In total, 100% of all residues were in permitted areas. There were no outliers in the dataset. Figure 2A,B depicts the 3D structure and Ramachandran graph values of DBP.

### 2.2. Binding Pocket Analysis

**Figure 3:**
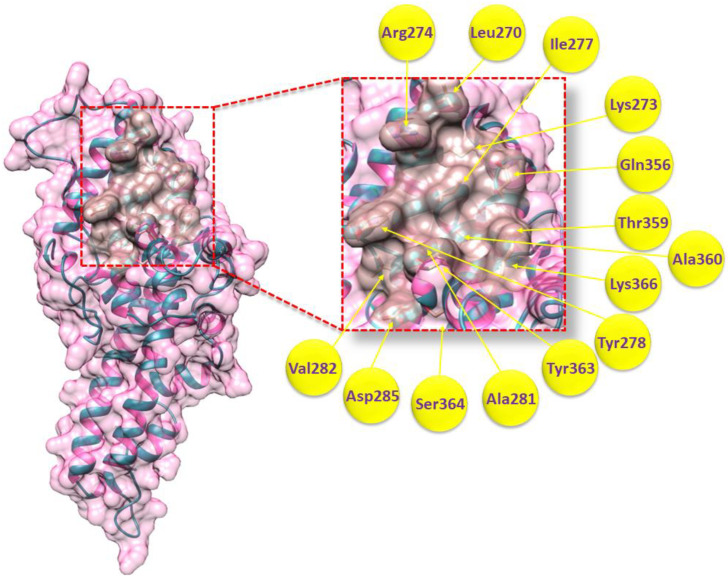
DBP’s binding pocket surface is emphasized in rosy brown color, whereas the rest of the protein surface appears pink. The ribbon color is dark cyan, while the interiors of the ribbon helix are identified with a hot pink tint. Furthermore, binding site residues that participate in the DBP–DARC complex are labeled with their amino acid number based on their position in the active binding site of the protein.

The DBP interacts with DARC on the surface of RBCs for *P. vivax* to invade RBCs. A significant opportunity for parasite control is provided by inhibiting this important interaction (Osii, 2022). Leu270, Lys273, Arg274, Ile277, Tyr278, Ala281, Val282, Asp285, Gln356, Thr359, Ala360, Tyr363, Ser364, and Lys366, which were chosen as the binding residues of *P. vivax* DBP (Figure 3), are previously published in a research paper [20]. These residues are crucial in the development of the DBP–DARC complex. Therefore, such residues are being studied further in molecular docking.

### 2.3. Licorice (Ligands) Preparation

**Figure 4:**
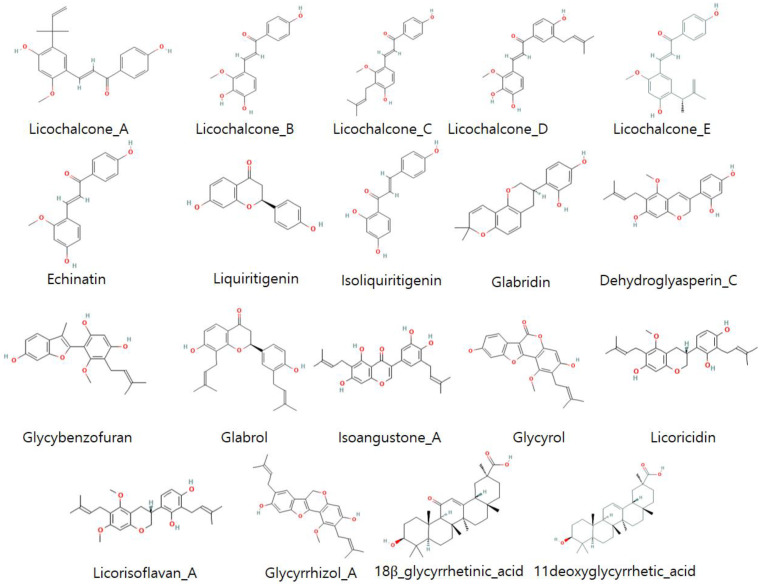
The 2D structure analysis of licorice compounds.

Licorice compounds are most prevalently used to combat many types of diseases such as cancer, viral infections, and parasitic infection. Their 3D models are downloaded and displayed in PyMOL and Discovery Studio for extensive 2D and 3D evaluation. Therefore, 19 licorice compounds (licochalcone_A, licochalcone_B, licochalcone_C, licochalcone_D, licochalcone_E, echinatin, liquiritigenin, isoliquiritigenin, glabridin, dehydroglyasperin_C, glycybenzofuran, glabrol, isoangustone_A, glycyrol, licoricidin, licorisoflavan_A, glycyrrhizol_A, 18β_glycyrrhetinic_acid, and 11deoxyglycyrrhetic_acid) were selected on the basis of their best structural conformation (Figure 4) and biological activity against various diseases (see Table 1) for our molecular docking studies.

### 2.4. Molecular Docking Analysis 

All docked complexes against DBP were examined independently and evaluated based on minimum energy values and ligand interaction patterns (Table 2). The top five licorice compounds (Licochalcone B, echinatin, licochalcone A, licochalcone E, and liquiritigenin) were ranked and chosen based on their lowest docking energies and ligand interaction patterns for further MD simulation analysis. The five ligands demonstrated the lowest docking energy and bound in the active region of the target protein. 

### 2.5. Interaction Analyses of the Top Five Ligands against DBP

The determination of the top five compounds that manifest the lowest molecular docking energy was carried out through binding mode analysis to see the exact interactions and blockage of the targeted protein’s active site.

### 2.6. Licochalcone B

**Figure 5:**
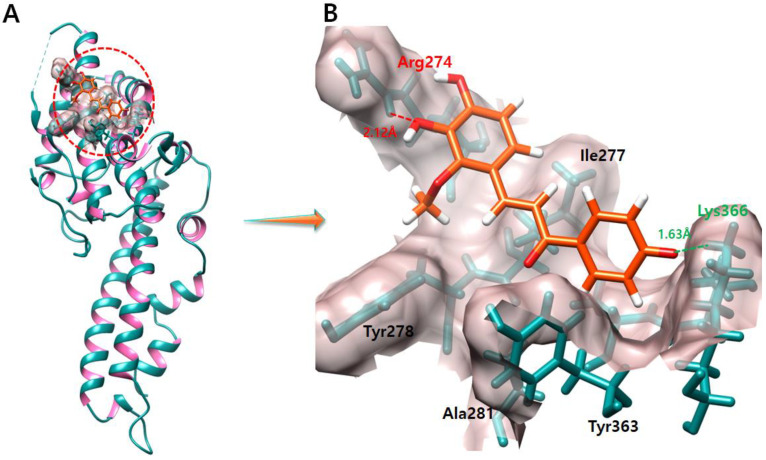
The figure (**A**) shows the licochalcone B–DBP complex. In figure (**B**) salt bridges and hydrogen bonds formed during the docking experiment are depicted in green and red color, respectively. One oxygen atom of daidzein forms a salt bridge and a hydrogen bond with Arg274 with a bond length of 2.12 Å. On the other hand, another oxygen atom forms a salt bridge with Lys366, and the length of the salt bridge is 1.63 Å.

The docking result of the licochalcone B–DBP depicts one hydrogen bond and one salt bridge at Arg274 and Lys366 residues. The oxygen atom of licochalcone B forms a hydrogen bond with Arg274 with a bond length of 2.12 Å, and the other oxygen atom of licochalcone B forms a salt bridge with Lys366 with a bonding distance of 1.63 Å (Figure 5). 

### 2.7. Echinatin

**Figure 6:**
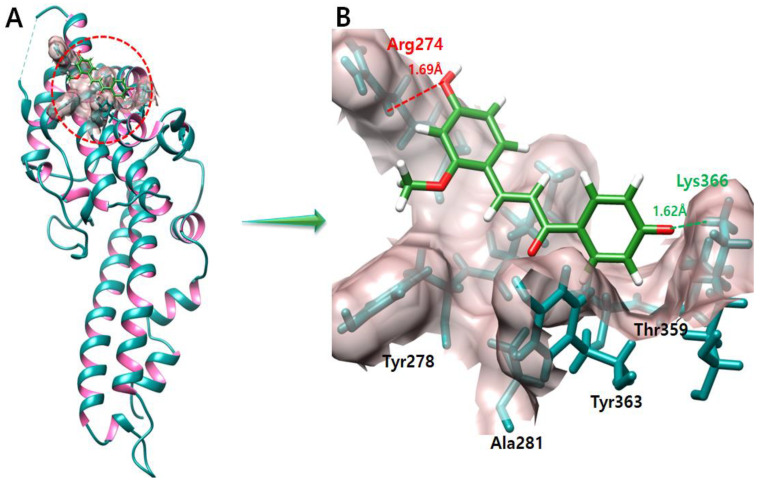
The figure (**A**) shows the echinatin and DBP complex. In figure (**B**) salt bridges and hydrogen bonds produced during the docking experiment are depicted in green and red color, respectively. One oxygen atom of echinatin forms a salt bridge with Lys366 with a length of 1.62 Å. On the other hand, another oxygen atom of echinatin forms a hydrogen bond with Arg274 with a length of 1.69 Å.

The docking result of the echinatin–DBP docked complex showed that one hydrogen bond and one salt bridge were formed by echinatin and Arg274 and Lys366 residues, respectively (Figure 6). The oxygen atom of echinatin forms a hydrogen bond with Arg274 with a bond length of 1.69 Å, and the other oxygen atom of echinatin forms a salt bridge with Lys366 with a bond length of 1.62 Å.

### 2.8. Licochalcone A

**Figure 7:**
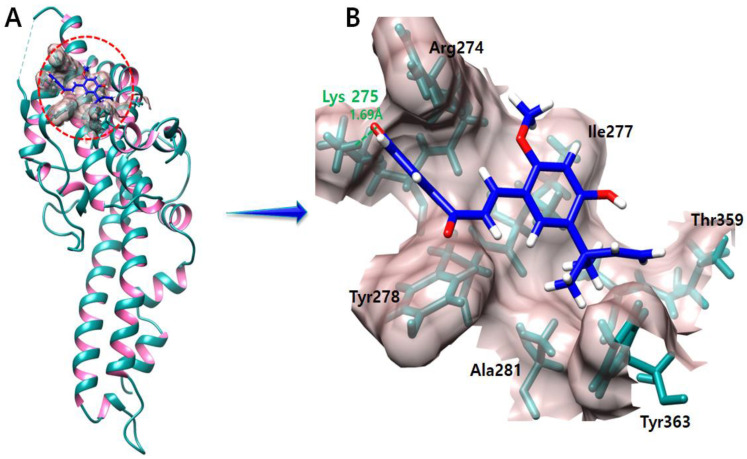
The licochalcone A–DBP complex is depicted in this figure (**A**). In figure (**B**) salt bridge produced during the docking experiment is shown in green color. An oxygen atom forms a salt bridge with the Lys275 with a length of 1.69 Å.

The binding interaction results of the licochalcone A–DBP docked complex manifest that the oxygen atom of licochalcone A forms a salt bridge with Lys275 with a bond length of 1.69 Å (Figure 7). Furthermore, the other interacting amino acids were also depicted in the graphical representation.

### 2.9. Licochalcone E

**Figure 8:**
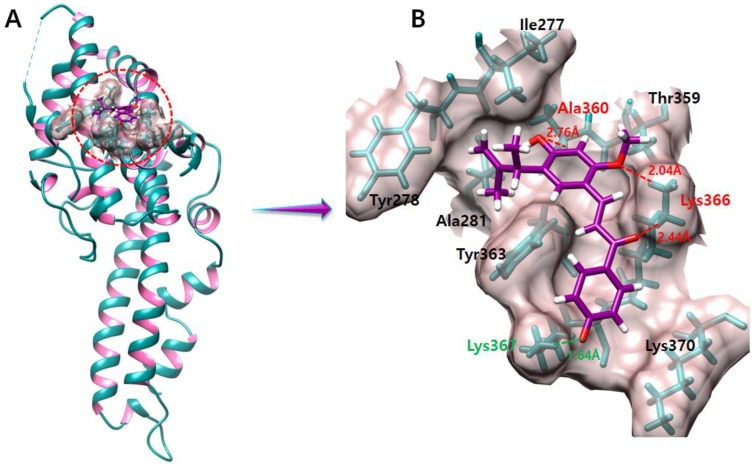
The figure (**A**) shows the licochalcone E and DBP complex. The salt bridges and hydrogen bonds produced during our docking experiment are shown in green and red color, respectively in figure (**B**).

The complex of licochalcone E–DBP demonstrates one hydrogen bond and one salt bridge with Ala360, lys366, and Lys367 residues (Figure 8). The oxygen atom of licochalcone E forms a hydrogen bond with Ala360 with a bond length of 2.76 Å. Another two oxygen atoms of licochalcone E produce two hydrogen bonds with the same Lys366 with bond lengths of 2.04 Å and 2.44 Å. Furthermore, another oxygen atom of licochalcone E makes a salt bridge with Lys366 with a length of 1.64 Å.

### 2.10. Liquiritigenin

**Figure 9:**
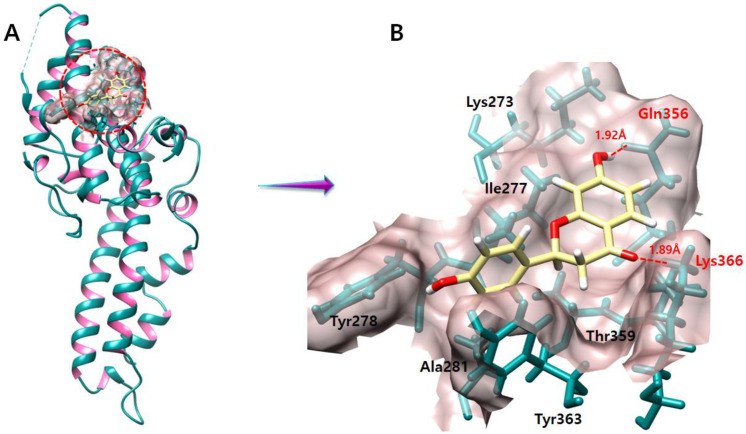
The figure (**A**) shows the liquiritigenin and DBP complex. In figure (**B**) the hydrogen bonds are represented in red color and the bonding residues are also labeled in red color. An oxygen atom of pulchellidine forms a salt bridge with Lys366 with a length of 1.68 Å.

The liquiritigenin molecule forms two hydrogen bonds with Lys366 and Gln356 residues. The oxygen atom of liquiritigenin forms a hydrogen bond with Lys366 with a length of 1.89 Å, while another oxygen atom of liquiritigenin forms a hydrogen bond with Gln356 with a length of 1.92 Å (Figure 9) in the active region of the targeted protein.

### 2.11. Molecular Dynamic Simulation

To test the stability of the best-docked complexes with the lowest binding energy conformation, the MD simulation approach was used. GROMACS, a Linux-based program, was utilized to conduct triplicates of 100 ns MD simulations [39]. The stability of licorice compounds in the binding region of *P. vivax* DBP were determined by RMSD analysis, binding mode analysis, and interaction energy analysis.

### 2.12. RMSD Analysis

**Figure 10:**
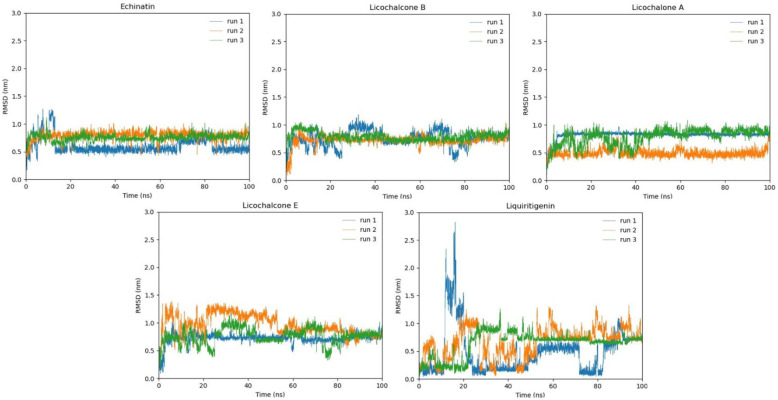
The graph exhibits the root mean square values of five selected compounds throughout a 100 ns MD simulation period. Three runs are indicated by different colors: run1 is indicated in blue color, run2 is exhibited in orange color, and run3 is determined by light green color. All three runs manifest RMSD values for 100 ns carried individually.

To evaluate the flexibility and overall stability of the docked complexes, 100 ns long MD simulations using GROMACS were conducted. The fluctuations of ligands inside the active site of the DBP protein were determined by root-mean-square-deviation (RMSD) from MD trajectories. Figure 10 shows the RMSD plots of licorice compounds against DBP protein during the 100 ns simulation. 

The licochalcone B molecule, which depicted the lowest molecular docking score and salt bridge formation in molecular docking studies, manifests approximately the same conformation in run2 and run3, while a little fluctuation in the graph can be seen in run1. The echinatin compound depicts the most stable RMSD values in run2 and run3, while, in run1, the graph shows that the RMS deviation point changes to σ = 0.7 from 70 ns to 83 ns in 100 ns MD simulation. The compound licochalcone A depicts the most stable RMSD in run1, while run2 also predicts a stable conformation. Meanwhile, run3 is a little fluctuating. The RMSD values of licochalcone E manifest stable RMSD in run1. Therefore, run2 and 3 are fluctuating at the start, but they are merging at the same point at 90 ns and become stable from 90 ns to 100 ns. The liquiritigenin compound, which exhibits high energy values in molecular docking, depicts the most fluctuating bar graph. In all three runs, the bar graph keeps fluctuating until the end, where the RMSD values remain stabilized to σ = 0.9 (Figure 10). 

### 2.13. Binding Modes Analysis after the MD Simulation

**Figure 11:**
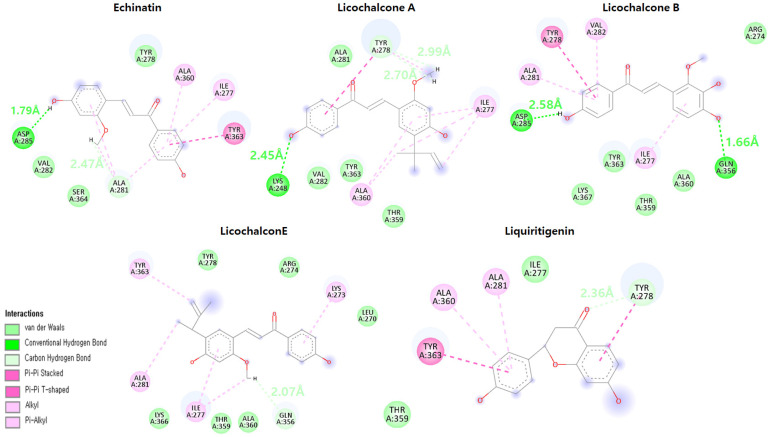
The figure depicts the interaction of licorice compounds in the active region of DBP. The conventional hydrogen bonds are predicted in green color, while carbon hydrogen bonds are depicted in light green color, and the hydrogen bonding distances are mentioned with respective colors.

The snapshots of all five complexes were obtained from 100 ns MD simulations, and the interactions were visualized using Discovery Studio and UCSF Chimera tools [40,41]. Licochalcone A, which showed only one hydrogen bond in molecular docking with Lys275, maintained one conventional hydrogen bond with Gln356 with a bond length of 2.11 Å and one carbon hydrogen bond with Ala360 with a bond length of 2.41 Å, which shows that the bounded ligand remained intact to the binding pocket throughout the 100 ns MD simulation experiment (Figure 11). Echinatin also maintained one conventional hydrogen bond with Asp285 with a bond length of 1.85 Å and the second carbon hydrogen bond with Val282 with a bond length of 2.61 Å. Echinatin also predicts a very stable RMS deviation graph and remains intact in the active region of the protein (Figure 11). 

Licochalcone B, which showed the lowest binding energy in molecular docking and the most stable RMSD values both in run1 and run2, maintained one conventional hydrogen bond with Gln356 with a bond length of 2.52 Å and a secure active binding site of the target protein (Figure 11), while licochalcone E and liquiritigenin, which predict high fluctuations in RMSD and higher energy values as compared to licochalcone A, licochalcone B, and echinatin in molecular docking, also exhibit good interaction with the target protein. Licochalcone E carries one conventional hydrogen bond and two carbon hydrogen bonds with DBP active region amino acids, while liquiritigenin has two conventional hydrogen bonds with the target protein with bond lengths of 1.96 Å and 3.02 Å.

### 2.14. MD Simulation Interaction Energy

**Figure 12:**
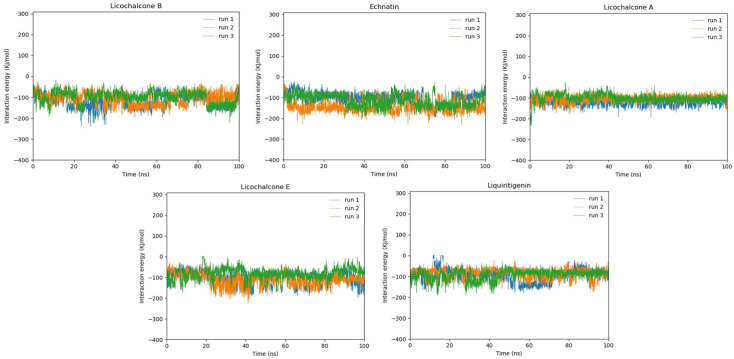
Bar graph depicting the stability of total interaction energy for licochalcone B, echinatin, licochalcone A, licochalcone E, and liquiritigenin for all three runs of 100 ns. Every run is indicated by a different color (run1—blue, run2—orange, run3—light green). The triplicates of every compound were predicted separately.

Along with RMSD and binding mode analysis, interaction energy calculations for all those five compounds against DBP were also carried out during 100 ns MD simulation of all three runs to examine the interaction energy score values. The interaction energy was analyzed in two forms, Coulombic interaction energy and Lennard-Jones interaction energy, and the sum of both energies was denoted by total interaction energy. The echinatin molecule showed the lowest interaction energy, at –119.7481. Meanwhile, licolchalcone B, which showed the lowest energy values in molecular docking studies, acquired a little more interaction energy than echinatin in MD simulation. Furthermore, licochalcone A, which showed the most stable RMSD value in run1, exhibited an average energy of –105.9473 for the 3 runs. Moreover, the licochalcone E and liquiritigenin molecules, which manifest high energy values in molecular docking and had higher RMS deviation rates than others, manifest the highest interaction energy in MD simulation interaction energy analysis (Table 3). Moreover, the total interaction energy of all these five compounds was also depicted in the bar chart, which predicts the most stable and similar plotting pattern for all five compounds in the respective three runs (Figure 12).

## 3. Discussion

*Glycyrrhiza glabra* L., also known as licorice, has shown potential therapeutic effects in the treatment of gastric ulcers, malaria, and hepatic disorders [16]. The results in this study demonstrate that licochalcone B, echinatin, and licochalcone A exhibit a good profile against *P. vivax* DBP. Discovery Studio was employed to dock 19 recently biologically active compounds against DBP. The molecular docking studies demonstrate that the ligands bind in the active region of DBP and block its active site by hindering the active site amino acid residues with the lowest molecular docking energies. The prediction of best compounds based on the docking score was recently found to be a non-promising approach [42]. Therefore, MD confirmations are required to predict the best fit against the receptor. The top five docked complexes based on their lowest docking energy were indulged in MD simulation analysis. The triplicate runs were carried out to check the stability of the compound with different starting valencies. All five compounds were keenly observed through RMSD, binding mode, and interaction energy analysis. The RMSD analysis manifests that licochalcone B, echinatin, and licochalcone A exhibit the most stable RMS deviation as compared to licochalcone E and liquiritigenin. Furthermore, the binding mode analysis was carried out to obtain the interacting amino acids and to confirm if the ligands bind with the active amino acids throughout 100 ns. The snapshots were taken at 100 ns, and interactions were analyzed using Discovery Studio. The binding mode analysis reveals that the ligands bind with the active amino acid residues until 100 ns, and conventional hydrogen bonds, carbon hydrogen bonds, and electrostatic forces were observed. Moreover, the MD interaction energy calculation was carried out using Coulomb and Lennard-Jones contributions, and the sum of both was mentioned as total interaction energy, while the average of three runs was calculated and exhibited in tabular form. The average MD interaction energy was also manifested in a graphical representation that depicts the almost similar energy patterns for all five compounds. 

The current study employed the particle mesh Ewald (PME) technique, a frequently used approach for computing long-range electrostatic interactions between charged particles in molecular dynamic simulations. Electrostatic interaction calculations are computationally costly, particularly in systems with a number of charged particles. The PME method provides an efficient way to calculate these long-range electrostatic interactions while maintaining high accuracy [43]. Although there are several other algorithms to calculate electrostatic interactions, currently, Reaction Field (RF) has been found to be less computational costly as compared to PME. In the RF approach, the electrostatic interaction energy between two charged particles is determined by combining the direct interaction energy and the reaction field energy. Using a cutoff distance beyond which the interaction is presumed to be negligible, the direct interaction energy is determined [44].

Computer-aided drug discovery (CADD) has shown to be an incredible resource in accelerating the development of epigenetic inhibitors by assisting in the selection, screening, designing, and optimizing of existing drugs, predicting their efficacy against new targets, identifying potential side effects, and improving pharmacokinetic properties, which leads to the discovery of novel compounds that can also be used to predict the properties of drug candidates and evaluate their effectiveness in silico before experimental testing [45,46]. Although in the least understood, novel, and complex cases, CADD is found to be struggling in many cases, the computationally predicted drugs have shown promising in vitro results, and they are being used against many diseases commercially. Captopril, Saquinavir, Zanamivir, Boceprevir, Nolatrexed, Rupintrivir, Aliskiren, Dorzolamide, and Oseltamivir are the drugs that were developed using CADD initially, and they have shown promising results against heart failure, human immunodeficiency virus (HIV), swine flu, hepatitis C virus (HCV), liver cancer, human rhinovirus (HRV), human renin, ocular hypertension, and influenza in vitro, with some of them being in clinical phase 3 trials [47]. Therefore, biological studies are necessary to confirm computational results. The examination of the binding inhibition efficacy of licorice compounds using ELISA assay and cell culture with purified DBP and DARC proteins is needed to validate the results. Furthermore, the biological activity of licorice compounds using human blood samples or animal models needs to be examined.

## 4. Methodology

### 4.1. Repossession of 4NUV from PDB

*P. vivax* DBP heterotetramer structure was obtained from the protein data bank (PDB) with PDBID 4NUV (www.rcsb.org (accessed on 15 February 2023)). 4NUV’s heterotetrameric structure was reduced into a single chain monomeric form and designated as the target protein (DBP). The target protein’s energy minimization was performed by utilizing Discovery Studio [40]. Furthermore, the DBP Ramachandran graph was evaluated using Discovery Studio [48]. The online web server VADAR 1.8 (VADAR) was used to obtain the protein architecture and statistical percentage values of helices, beta sheets, coils, and turns. 

### 4.2. Binding Site Assessment of DBP

The position of a ligand in the protein’s holo-structure most likely determines the binding pocket of the targeted protein and channels [49]. It is composed of certain amino acid residues that catalyze a reaction with that substrate (catalytic site) and residues that temporarily interact with the ligand (binding site) [50]. The active binding site residues are chosen from the data that have already been published [20] and acknowledged by Discovery Studio and the UCSF Chimera tool. Therefore, the binding site is specified by the current selection approach and the docking sphere constricted in the discovery studio to be constrained to our selected amino acid residues.

### 4.3. Licorice (Ligands) Preparation

Licorice compounds change the erythrocyte membrane at the concentration area where anti-plasmodial action is exhibited [51]. The ligand molecules are selected based on their recently reported biological activity (Table 1). The 3D structure of these 19 representative ligand molecules (licorice) were retrieved from PubChem and further minimized by visualizing in Discovery Studio and PyMOL. The representative ligands were also evaluated on the basis of their structures (2D, 3D) for molecular docking studies.

### 4.4. Molecular Docking Analysis Using Discovery Studio

Molecular docking is the most widely used method for evaluating the interactions and conformations of ligands with target proteins [52]. By using scoring functions, it is feasible to anticipate the connection strength or binding interaction between two molecules based on preferred orientation [53]. To perform molecular docking of licorice compounds against DBP, Discovery Studio’s “CDocker” approach was used. The binding pocket sphere values were adjusted as (X = _−12.1487, Y = _ 51.0354, and Z = _ 53.4578) and the radius value was adjusted as 7.4148 for improved conformational location in the active region of the target protein [20]. All of the compounds were docked against DBP individually using the default orientation and conformation 10, 10 correspondingly. Meanwhile, the top hits were chosen as 05. The lowest binding energy (Kcal/mol) values were used to assess the anticipated docked complexes. Discovery Studio (4.1) and UCSF Chimera 1.10.1 [41] were used to create a three-dimensional (3D) graphical depiction of the top five docked complexes.

### 4.5. Molecular Dynamic Simulation

The simulation methodology and parameters were obtained from previously published data [39] and extended to run the triplicates of 100 ns MD simulation experiments. The best complexes based on their molecular docking score and binding patron to the active region of the targeted protein were subjected to an MD simulation experiment. The GROMACS tool (version 2019.3) was used under the Linux operating system to examine the structural behavior of protein and ligand complexes [54]. The CHARMM-GUI server’s solution builder protocol (www.charm-gui.org (accessed on 20 March 2023)) was used to generate the CHARMM36 force field, and the same interface was used to construct input files for MD simulations in GROMACS [55]. TIP3P solutions were used to solvate the existing model into a periodic, cubic box that was expanded by 10 Å beyond each protein atom. Counter ions were added until the system was neutralized. The Verlet cutoff technique with 10 Å was employed for electrostatic and van der Waals interactions, while the LINCS algorithm was applied to restrict bonds. Furthermore, the particle mesh Ewald (PME) technique was used to calculate the electrostatic interactions. The solvated systems were subjected to the steepest descent energy minimization. Following that, the systems proceeded through two rounds of equilibration. Systems were first brought into equilibrium under the NVT condition. During the NVT equilibration phase, the number of particles, volume, and temperature are kept constant; then, under the NPT condition, during the NPT equilibration phase, the number of particles, pressure, and temperature are kept constant. Thus, the system can exchange energy and particles with the thermostat and barostat to maintain a constant temperature and pressure, respectively. Therefore, the CHARMM-GUI includes a Python script for converting GROMACS topology (top) and parameter (itp) files for MD simulations in GROMACS. To execute production dynamics in GROMACS, a 2 fs time step was used, and coordinates were written to a file every picosecond for analysis.

## 5. Conclusions

In the blood stage, *P. vivax* DBP invades red blood cells through specific types of receptors present on their surface (DARC). To block the DBP–DARC complex formation, DARC binding residues of DBP are subjected to blockage with the screened library of licorice compounds. Therefore, licorice compounds showed significant compatibility against *P. vivax* DBP. Licochalcone B, echinatin, licochalcone A, licochalcone E, and liquiritigenin exhibited the lowest docking energy values in molecular docking analysis and bind to the active region of the protein in binding analysis. Furthermore, these five ligands were subjected to MD simulation for the analysis of the stability of docked complexes, and the triplicates of MD simulation were carried out for each compound. Licochalcone B, echinatin, and licochalcone A blocked the active site of DPB protein with the lowest interaction energy both in molecular docking and MD simulation and higher stability in RMSD analysis, followed by licochalcone E and liquiritigenin. Therefore, licochalcone B, echinatin, and licochalcone A compounds appear to be promising against *P. vivax* DBP, which binds to the active region of DBP and remains intact throughout the entire study. In conclusion, licorice compounds should be investigated as a promising possibility for inhibiting *P. vivax* invasion into human RBCs via DBP–DARC interaction. Furthermore, modifying licorice compounds might be a promising approach for future prospects to obtain more efficient ligands against *P. vivax* DBP.

## Figures and Tables

**Table 1:** Recent biological activities of selected compounds.

Sr No	Ligand Name	Activity	Reference
1	Licochalcone B	Antitumor, anti-inflammatory, antiviral	[21,22,23]
2	Echinatin	Antiviral	[22,24]
3	Licochalcone A	Antiviral, antimicrobial, immunoregulatory	[21,25]
4	Licochalcone E	Antiviral, antimicrobial	[25]
5	Liquiritigenin	Antiviral, antimicrobial, immunoregulatory	[21,25]
6	Licochalcone C	Anticancer	[26]
7	Isoliquiritigenin	Antiviral	[27]
8	Glabridin	Antiviral	[28,29]
9	Dehydroglyasperin C	Hepatoprotective	[30]
10	Licochalcone D	Antiviral, antioxidant	[31]
11	Glycygenzofuran	Diabetes mellitus, obesity	[32]
12	Glabrol	Hepatoprotective	[25]
13	Isoangustone A	Adenocarcinoma	[33]
14	Glycyrol	Antiviral	[29]
15	Licoricidin	Antiviral	[34]
16	Licorisoflavan A	Antimicrobial	[35]
17	Glycyrrhizol A	Anticaries, antimicrobial	[36,37]
18	18β Glycyrrhetinic acid	Antiviral, antimicrobial	[25]
19	11Deoxyglycyrrhetic acid	Antiviral	[38]

**Table 2:** Docking energy table of the screened flavonoids calculated by CDocker.

No	Ligand Name	CDocker Energy	CDocker Interaction Energy
1	Licochalcone B	−40.646	−47.6473
2	Echinatin	−36.4715	−44.7142
3	Licochalcone A	−33.3302	−52.3068
4	Licochalcone E	−30.6573	−54.8755
5	Liquiritigenin	−22.072	−27.4566
6	Licochalcone C	−20.0702	−52.0653
7	Isoliquiritigenin	−14.967	−26.2369
8	Glabridin	−11.7479	−31.5265
9	Dehydroglyasperin C	−5.66293	−48.4455
10	Licochalcone D	−1.45512	−33.7997
11	Glycygenzofuran	6.02888	−34.8505
12	Glabrol	12.2899	−37.0889
13	Isoangustone A	12.5396	−35.0905
14	Glycerol	18.4799	−32.3047
15	Licoricidin	20.9019	−36.0512
16	Licorisoflavan A	25.2122	−35.4716
17	Glycyrrhizol A	35.3237	−33.7042
18	18β Glycyrrhetinic acid	43.0477	−38.4474
19	11Deoxyglycyrrhetic acid	51.5132	−38.6611

**Table 3:** The MD simulation interaction energy of the top five compounds was predicted in the table.

Ligand Name	Total Energy			Average Energy
	R1	R2	R3	
Licochalcone B	–105.8469	–116.8744	–99.7185	–107.4799
Echinatin	–102.3061	–143.673	–113.2652	–119.7481
Licochalcone A	–112.8598	–104.1913	–100.7909	–105.9473
Liquiritigenin	–84.437	–92.9508	–93.1907	–90.1928
Licochalcone E	–110.7497	–101.6259	–81.5792	–97.9849

## Data Availability

All the data are available in the manuscript.
